# The Editor's Letter-Box

**Published:** 1921-01-01

**Authors:** Hy. Eason

**Affiliations:** The London Lock Hospitals, Harrow Road and Dean Street, W.


**The Editor's Letter Box—(*continued*).**

*To the Editor of* The Hospital.

Sir,—I write to say that I cordially agree with what Mr. Glenton-Kerr says in the last issue of The Hospital. There appears to be a tendency to forget the excellent work of the Hospital Saturday Fund in London. As a hospital secretary, with an experience of some years in the provinces, I feel that to make the London Hospital Fund a success we must do all we can to help its Council, and aid it in its endeavours to concentrate all workmen's and working women's contributions into its coffers. In the Provinces parties of collectors and works representatives visit the voluntary hospitals, and are able to see the work at first hand, and thus their interest in the voluntary system is increased, and they can speak with authority on the need for increasing the amount annually collected and distributed. I look to the Saturday Fund to greatly increase its grants to the hospitals in the future; therefore let us not create amongst the working classes new collecting agencies, just when the Saturday Fund needs all the help we can give it.

I am, Sir, your Obedient Servant,

Hy. Eason.

The London Lock Hospitals,
Harrow Road and Dean Street, W.

### Housing the Nursing Staffs.

*To the Editor of* The Hospital.

